# Supplementary material for: Structural basis of meiotic telomere attachment to the nuclear envelope by MAJIN-TERB2-TERB1
Source: Nat Commun. 2018 Dec 17;9:5355. doi: 10.1038/s41467-018-07794-7 (PMC6297230; doi:10.1038/s41467-018-07794-7)
Supplement: Supplementary file 1 — Supplementary Information [file 41467_2018_7794_MOESM1_ESM.pdf]

Supplementary information

Structural basis of meiotic telomere attachment to the nuclear envelope by MAJIN-TERB2-TERB1

J. M. Dunce, A.E. Milburn et al.

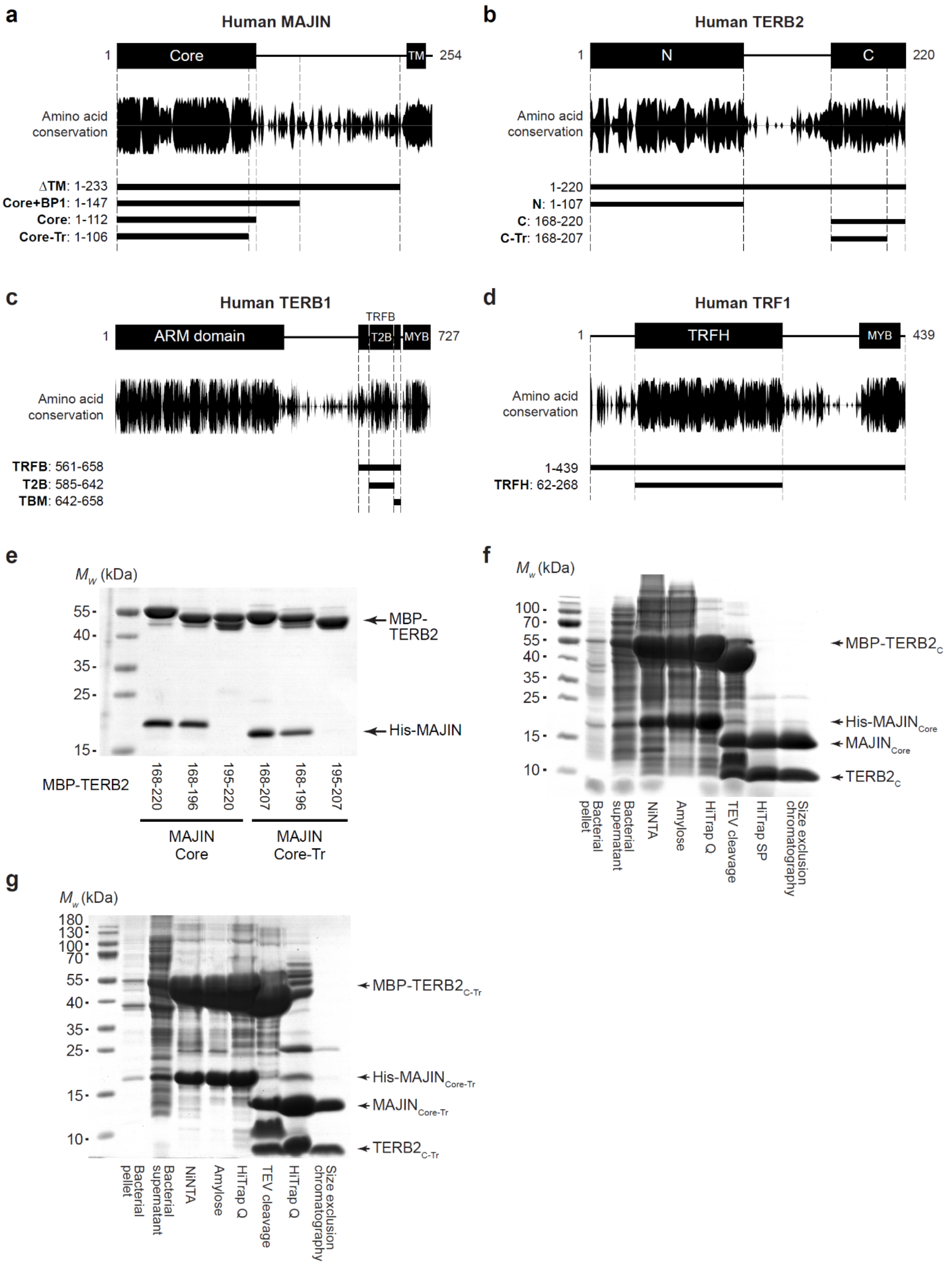

Supplementary Figure 1

Summary of meiotic telomere complex component constructs and the MAJIN-TERB2 complex

(**a**-**d**) Sequence schematics of human meiotic telomere complex components (**a**) MAJIN, (**b**) TERB2 and (**c**) TERB1, and (**d**) shelterin component TRF1. Domain structure and amino acid conservation are aligned with the sequence boundaries of constructs used in this study. (**e**-**g**) Identification of a direct biochemical interaction

between MAJIN and TERB2. (**e**) Recombinant co-expression and amylose pull-down of His-MAJIN (Core: amino acids 1-112; Core-Tr: amino acids 1-106) with MBP-TERB2 amino acids 168-220, 168-196, 195-220, 168-207 and 195-207. (**f**,**g**) Recombinant co-expression and co-purification of (**f**) $MAJIN_{Core}$-$TERB2_{C}$ (amino acids 1-112; 168-220) and (**g**) $MAJIN_{Core\text{-}Tr}$-$TERB2_{C\text{-}Tr}$ (amino acids 1-106; 168-207) through Ni-NTA, amylose, and anion exchange chromatography, following by TEV cleavage to remove N-terminal expression tags, with subsequent cation or anion exchange (as indicated) and size exclusion chromatography. Source data are provided as a Source Data file.

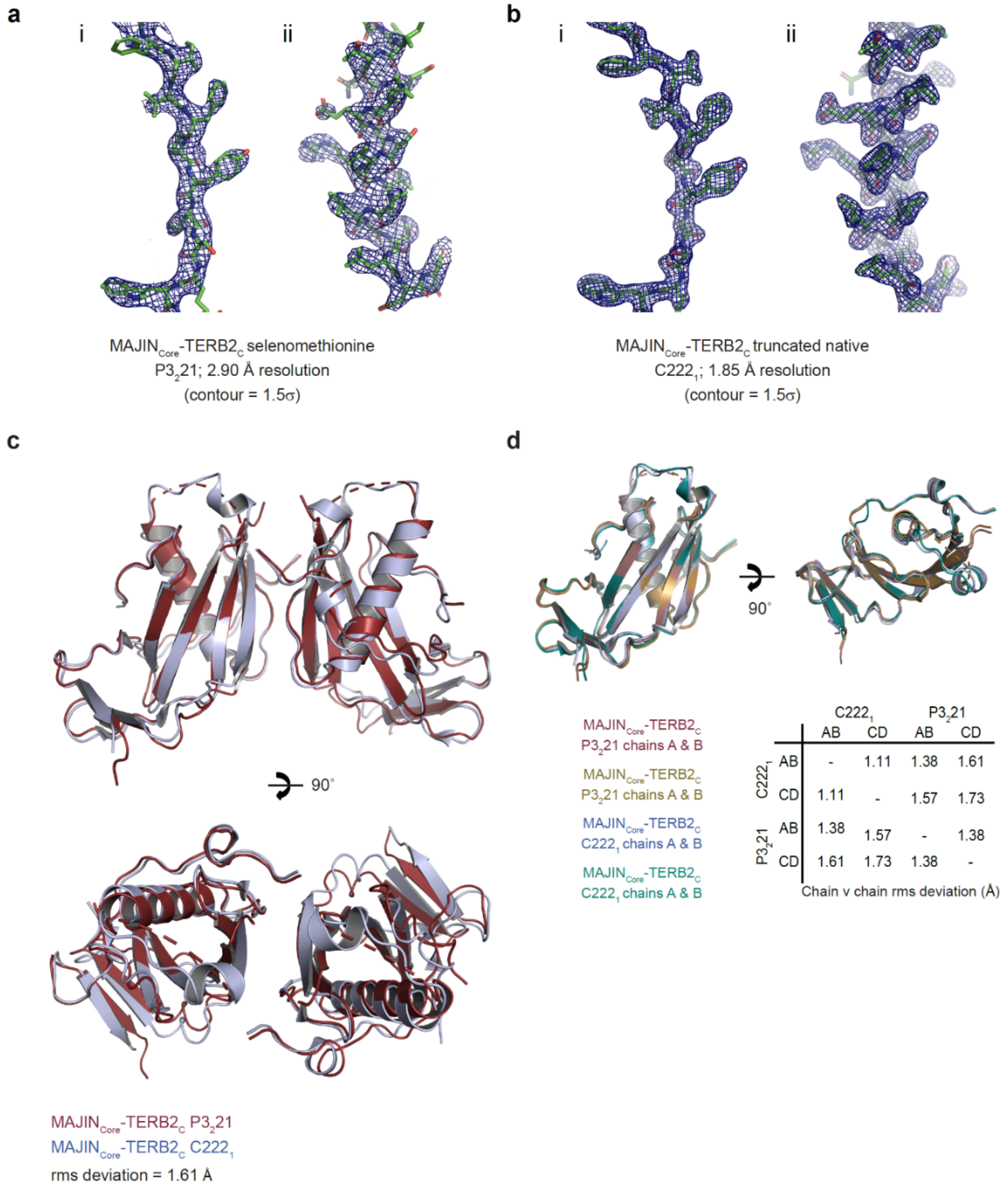

Supplementary Figure 2

Structure solution of MAJIN$_{Core}$-TERB2$_C$ by X-ray crystallography

(**a**,**b**) 2Fo-Fc electron density maps contoured at 1.5σ and superimposed on the refined crystallographic models for (**a**) MAJIN$_{Core}$-TERB2$_C$ (amino acids 1-112; 168-220) seleno-methione derivative in P3$_2$21 spacegroup at 2.90 Å resolution and (**b**) MAJIN$_{Core}$-TERB2$_C$ truncated (amino acids 1-106; 168-207) in C222$_1$ spacegroup at 1.85 Å resolution. Identical regions of both structures are displayed, including examples of (**i**) β-strand and (**ii**) α-helical secondary structure. Crystal structures were solved by single wavelength anomalous diffraction of selenium atoms and molecular replacement using a single MAJIN chain of the former structure, respectively. (**c**) Superposition of the MAJIN$_{Core}$-TERB2$_C$ P3$_2$21 (red) and C222$_1$ (light blue) crystal structures with an rms deviation of 1.61 Å. (**d**) Superposition of the two unique MAJIN-TERB2 protomers (chains AB and CD) of the MAJIN$_{Core}$-TERB2$_C$ P3$_2$21 (red and gold) and C222$_1$ (blue and green) crystal structures; rms deviation values between chains are shown.

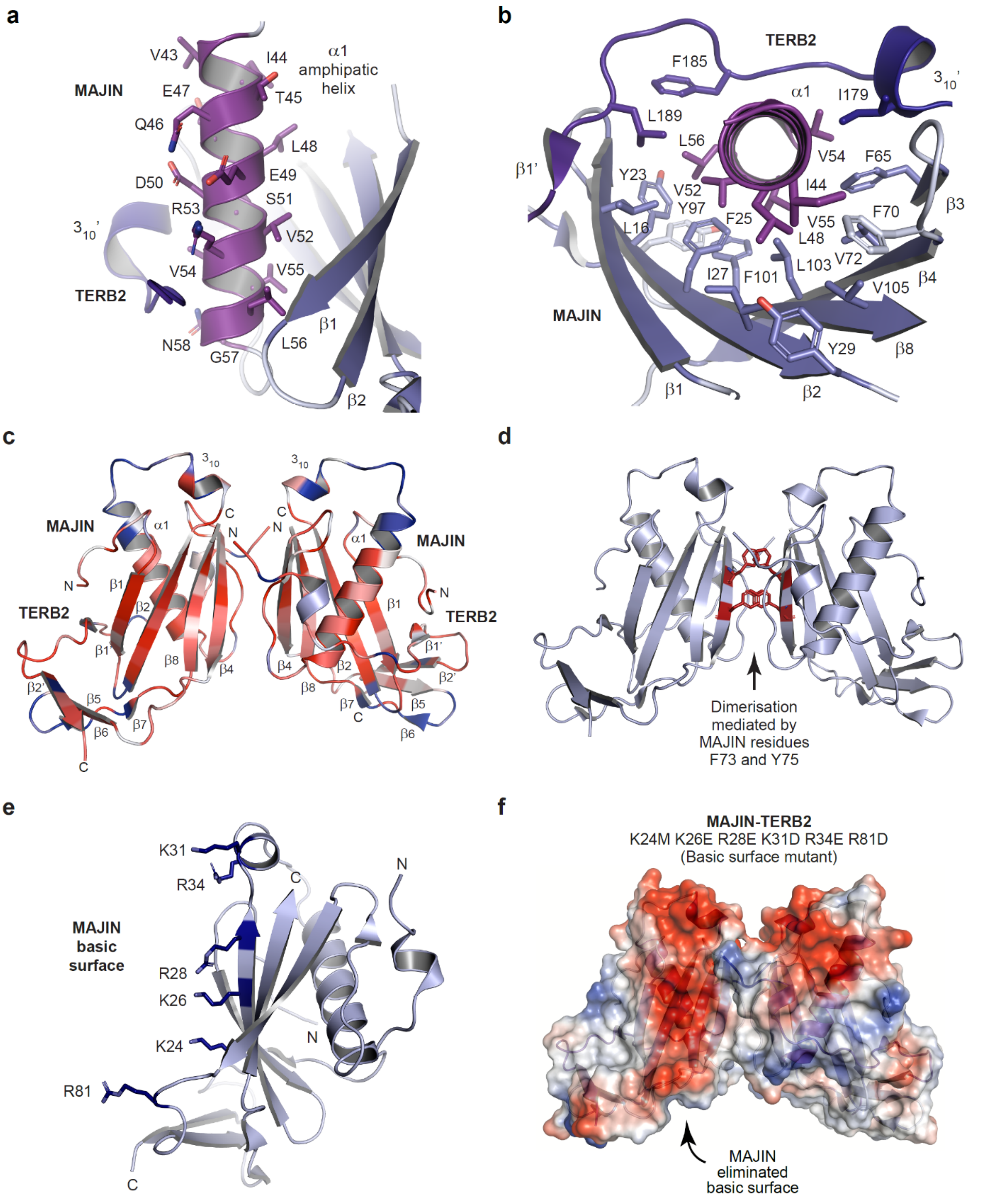

Supplementary Figure 3

Structural details of $\text{MAJIN}_{\text{Core}}$-$\text{TERB2}_{\text{C}}$

(**a**,**b**) The MAJIN central α-helix is amphipathic, formed of a hydrophobic surface that interacts with the internal surface of the grasping β-sheet and a hydrophilic surface that is largely solvent exposed. The latter surface contains a strip of hydrophobic residues towards its C-terminal end to enable its interaction with the

encircling TERB2 chain. (**c**) $MAJIN_{Core}$-$TERB2_C$ structure coloured according to amino acid conservation (red - highly conserved; blue - poorly conserved), indicating that the basic surface of the grasping β-sheet and the dimerization interface are highly conserved. (**d**) MAJIN residues F73 and Y75 shown within the $MAJIN_{Core}$-$TERB2_C$ structure to highlight their essential role at the dimerization interface. (**e**) A single $MAJIN_{Core}$-$TERB2_C$ protomer showing MAJIN residues K24, K26, R28, K31, R34 and R81, which constituent its basic surface. (**f**) Surface electrostatic potential of $MAJIN_{Core}$-$TERB2_C$ upon introduction of mutation K24M K26E R28E K31D R34E R81D, demonstrating elimination of the MAJIN basic surface (red - electronegative; blue - electropositive).

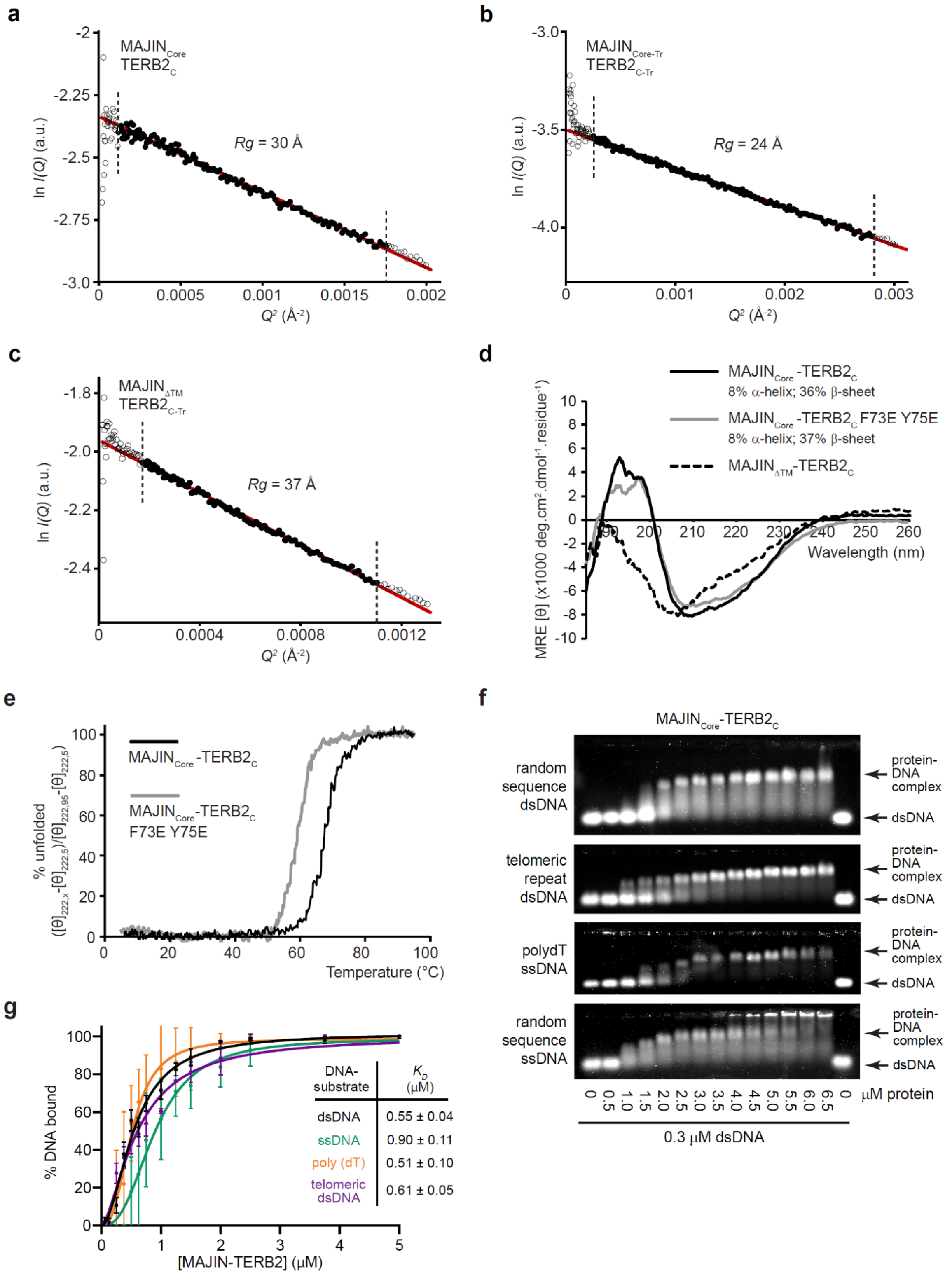

Supplementary Figure 4

Solution structure and DNA binding of MAJIN$_{Core}$-TERB2$_C$

(**a**-**c**) SAXS Guinier analysis to determine the radius of gyration (*Rg*); the Guinier regions are shown in black, demarcated by vertical dashed lines, with linear fits shown in red (*Q.Rg* values were < 1.3). (**d**) Far UV CD

spectra and (**e**) CD thermal denaturation of MAJIN$_{Core-}$-TERB2$_C$ wild type (black) and MAJIN mutant F73E Y75E (grey), and MAJIN$_{\Delta TM}$-TERB2$_C$ (black, dashed). (**d**) Secondary structure composition was estimated for MAJIN$_{Core-}$-TERB2$_C$ WT and F73E Y75E through deconvolution of spectra with data fitted at normalised rms deviation values of 0.061 and 0.063, respectively. (**e**) Thermal denaturation was recorded for MAJIN$_{Core-}$-TERB2$_C$ WT and F73E Y75E as % unfolded based on the helical signal at 222 nm; melting temperatures were estimated at 66°C and 59°C, respectively. (**f**) EMSA analysing the ability of MAJIN$_{Core}$-TERB2$_C$ to interact with a random sequence dsDNA (57 base pair substrate), telomere repeat dsDNA (54 base pair substrate), poly(dT) ssDNA (100 base substrate) and random sequence ssDNA (90 base substrate). Gel images are representative of at least three replicate EMSAs. (**g**) Quantification of MAJIN$_{Core}$-TERB2$_C$ binding to random dsDNA (black), ssDNA (green), poly(dT) ssDNA (yellow) and telomeric dsDNA (purple) through densitometry of EMSAs performed using 25 nM (per molecule) FAM-labelled DNA constructs. Plots and apparent K_D values were determined by fitting data to the Hill equation; error bars indicate standard error, n=3 EMSAs. Source data are provided as a Source Data file.

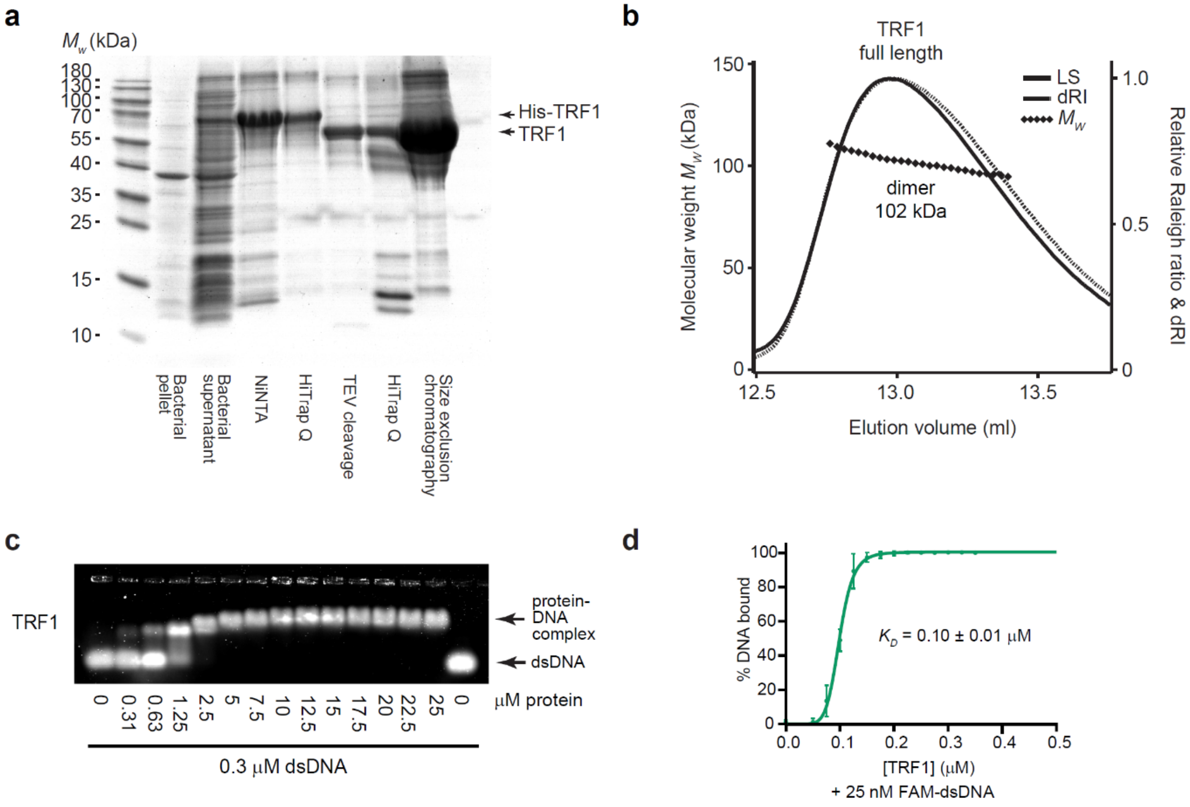

Supplementary Figure 5

Purification and biophysical analysis of full-length TRF1

(**a**) Recombinant expression and purification of TRF1 (1-439) through Ni-NTA, anion exchange chromatography, TEV cleavage to remove the N-terminal His-tag, with subsequent anion exchange and size exclusion chromatography. (**b**) SEC-MALS analysis of TRF1 demonstrating a 102 kDa homodimer (theoretical dimer – 100 kDa). (**c**) EMSA analysing the ability of TRF1 (full length) to interact with linear dsDNA. Gel images are representative of at least three replicate EMSAs. (**d**) Quantification of DNA-binding by $MAJIN_{Core}$-$TERB2_C$ through densitometry of EMSAs performed using 25 nM (per molecule) FAM-labelled dsDNA. Plots and apparent K_D values were determined by fitting data to the Hill equation; error bars indicate standard error, n=3 EMSAs. Source data are provided as a Source Data file.

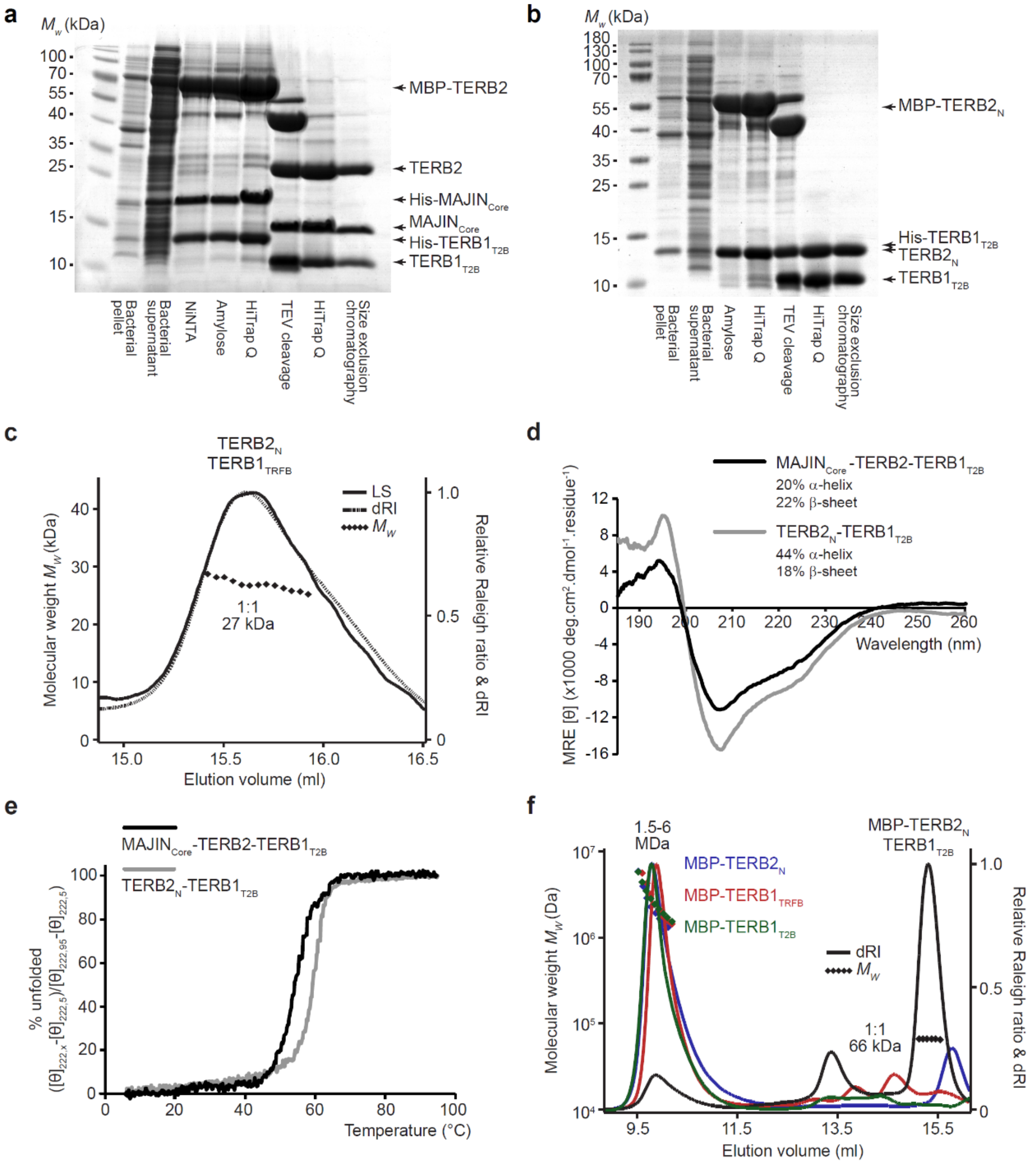

Supplementary Figure 6

Purification and biophysical analysis of MAJIN$_{Core}$-TERB2-TERB1$_{T2B}$ and TERB2$_{N}$ -TERB1$_{T2B}$

(**a**,**b**) Recombinant co-expression and co-purification of (**a**) MAJIN$_{Core}$-TERB2-TERB1$_{T2B}$ (1-112, 1-220, 585-642) and (**b**) TERB2$_{N}$-TERB1$_{T2B}$ (1-107, 585-642) through Ni-NTA, amylose, and anion exchange chromatography, followed by TEV cleavage to remove N-terminal expression tags, with subsequent anion exchange and size exclusion chromatography. Source data are provided as a Source Data file. (**c**) SEC-MALS analysis revealing that TERB2$_{N}$-TERB1$_{TRFB}$ is a 27 kDa 1:1 complex (theoretical 1:1 – 24 kDa). (**d**) Far UV CD spectra and (**e**) CD thermal denaturation of MAJIN$_{Core}$-TERB2-TERB1$_{T2B}$ (black) and TERB2$_{N}$-TERB1$_{T2B}$ (grey). (**d**) Secondary structure composition was estimated through deconvolution of spectra with data fitted at normalised rms deviation values of 0.026 and 0.011, respectively. (**e**) Thermal denaturation was recorded as % unfolded based on the helical signal at 222 nm; melting temperatures were estimated at 55°C and 60°C, respectively. (**f**) SEC-MALS analysis. In isolation, MBP-TERB2$_{N}$ (blue), MBP-TERB1$_{TRFB}$ (red) and MBP-TERB1$_{T2B}$ (green) form aggregates of 1.5-6 MDa, whereas a complex between MBP-TERB2$_{N}$ and TERB1$_{T2B}$ (black) forms a 66 kDa 1:1 heterodimer (theoretical 1:1 – 68 kDa).

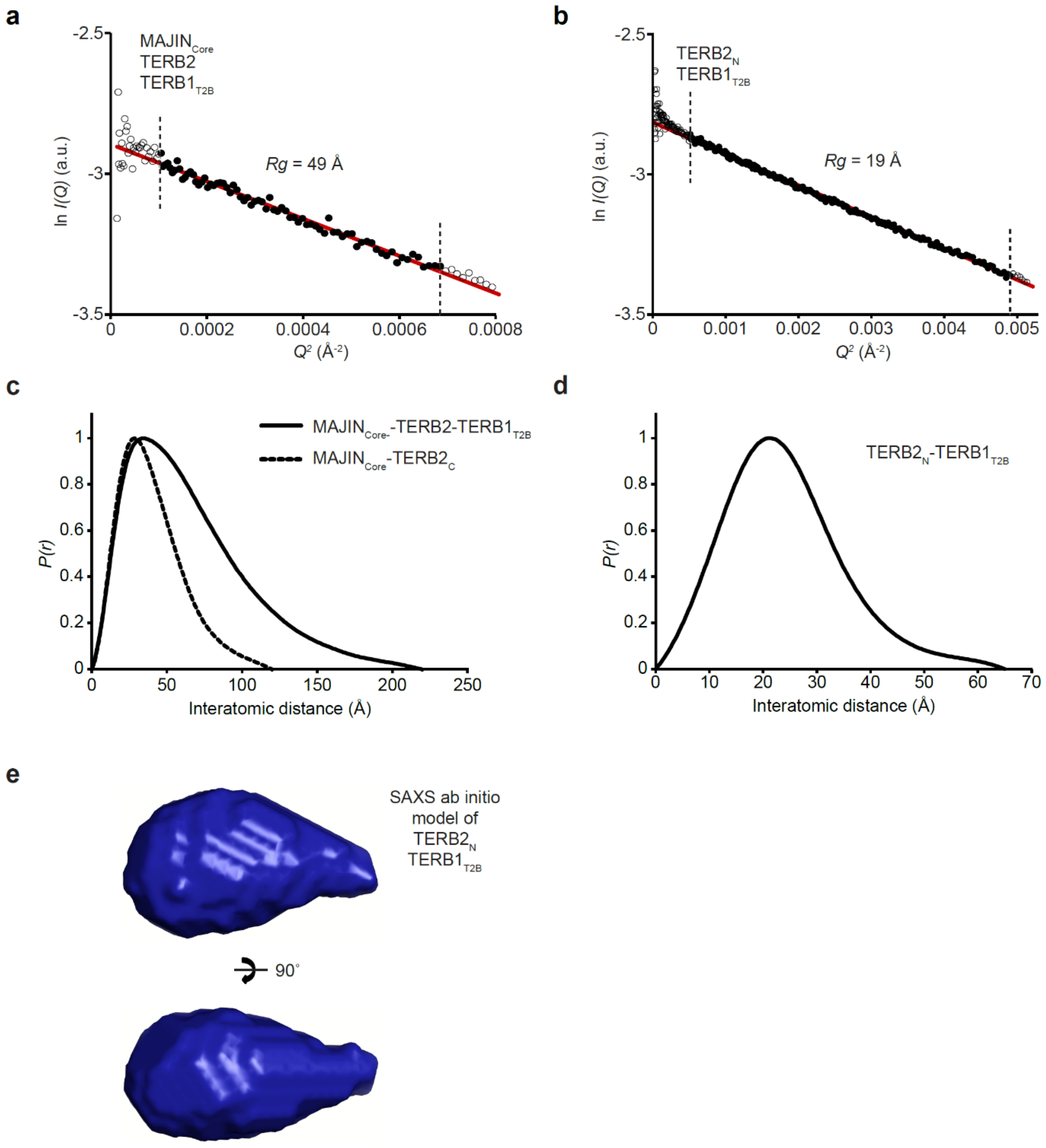

Supplementary Figure 7

SAXS solution structures of MAJIN$_{Core}$-TERB2-TERB1$_{T2B}$ and TERB2$_N$ -TERB1$_{T2B}$

(**a**-**c**) SEC-SAXS analysis of MAJIN$_{Core}$-TERB2-TERB1$_{T2B}$ and TERB2$_N$ -TERB1$_{T2B}$. (**a**-**b**) SAXS Guinier analysis to determine the radius of gyration (*Rg*) of MAJIN$_{Core}$-TERB2-TERB1$_{T2B}$ and TERB2$_N$ -TERB1$_{T2B}$; the Guinier regions are shown in black, demarcated by vertical dashed lines, with linear fits shown in red (*Q.Rg* values were < 1.3). (**c**-**d**) SAXS *P(r)* distributions of (**c**) MAJIN$_{Core}$-TERB2-TERB1$_{T2B}$ (black, solid), MAJIN$_{Core}$-TERB2$_C$ (black, dashed) and (**d**) TERB2$_N$-TERB1$_{T2B}$, showing maximum dimensions of 220 Å, 120 Å and 65 Å, respectively. Their real space *Rg* values of 54 Å, 32 Å and 19 Å closely match their Guinier analysis *Rg* values of 49 Å, 30 Å and 19 Å, respectively. (**e**) SAXS ab initio model of TERB2$_N$-TERB1$_{T2B}$. A filtered averaged model was generated from 30 independent DAMMIF runs, with NSD=0.560 (± 0.049) and reference model χ^2=1.40.

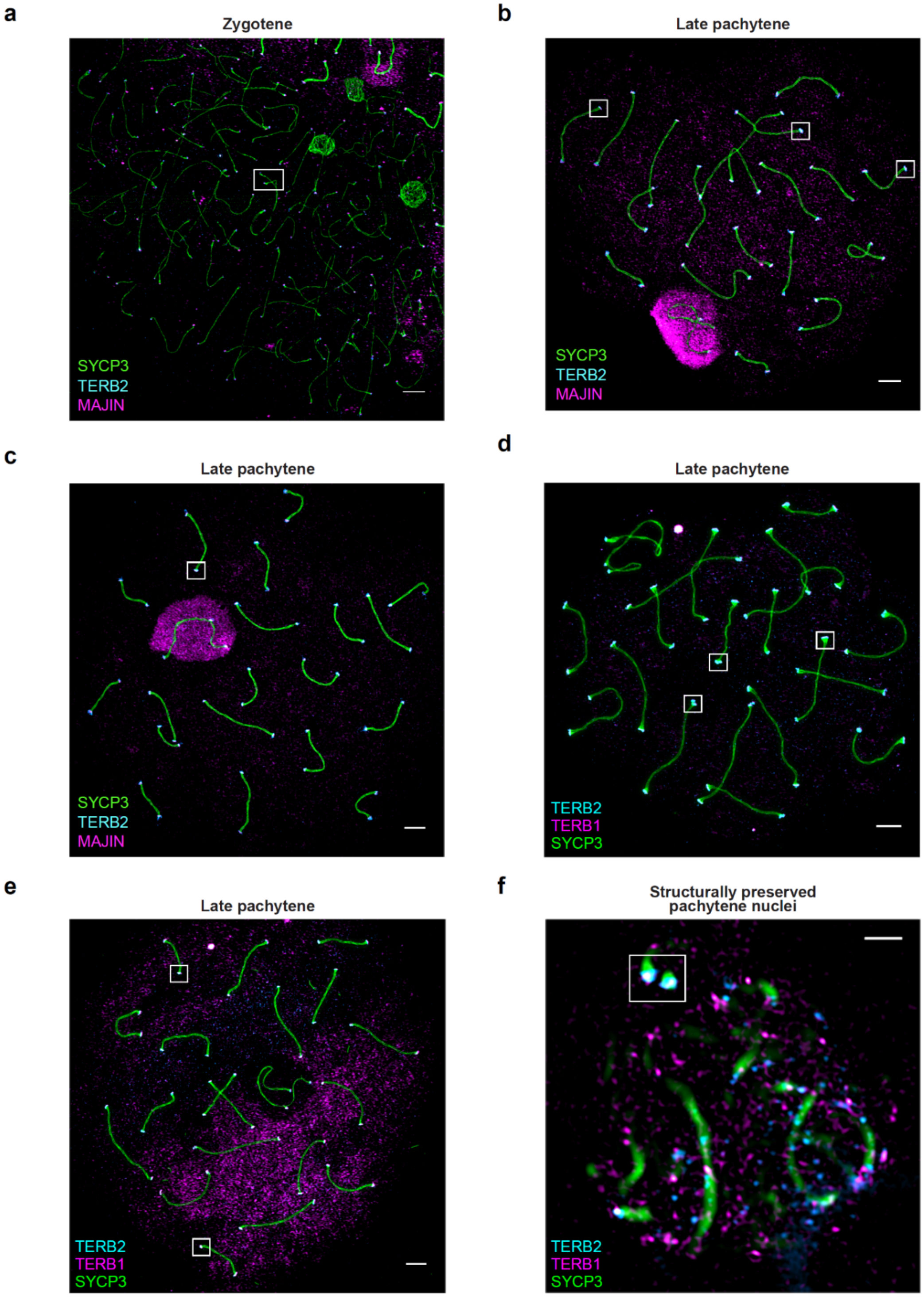

Supplementary Figure 8

Wide field SIM images of the meiotic telomere complex

Structured illumination microscopy of (**a**) mouse zygotene spermatocyte chromosome spreads, (**b**-**e**) mouse pachytene spermatocyte chromosome spreads and (**f**) structurally preserved mouse pachytene nuclei stained with anti-SYCP3 (green), anti-TERB2 (cyan) and (**a**-**c**) anti-MAJIN (magenta) or (**d**-**f**) anti-TERB1 (magenta). Scale bars, (**a**-**e**) 3 µm and (**f**) 5 µm. White squares represent the telomere ends used for display and analysis in (**a**) Fig. 5e, (**b**-**e**) Fig. 5f and (**f**) Fig. 5g.

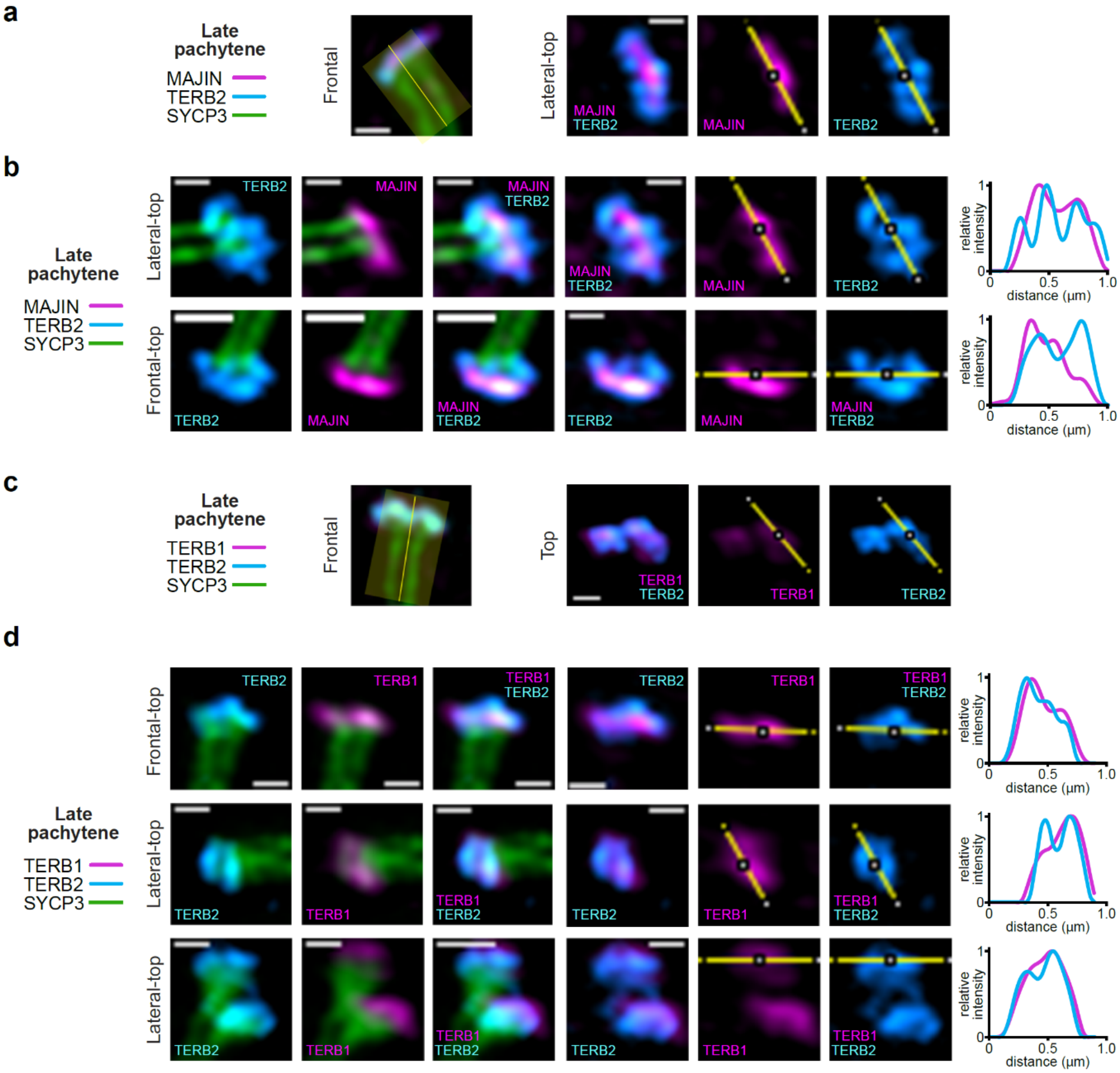

Supplementary Figure 9

SIM analysis of the meiotic telomere complex

Structured illumination microscopy of mouse pachytene spermatocyte chromosome spreads stained with anti-SYCP3 (green), anti-TERB2 (cyan) and (**a**,**b**) anti-MAJIN (magenta) or (**c**,**d**) anti-TERB1 (magenta). Scale bars, 0.3 µm. (**a**,**c**) Analyses relating to plots shown in Fig. 5e. (**b**,**d**) Additional orientations with normalised intensity-distance plots, relating to SIM data shown in Fig. 5e.

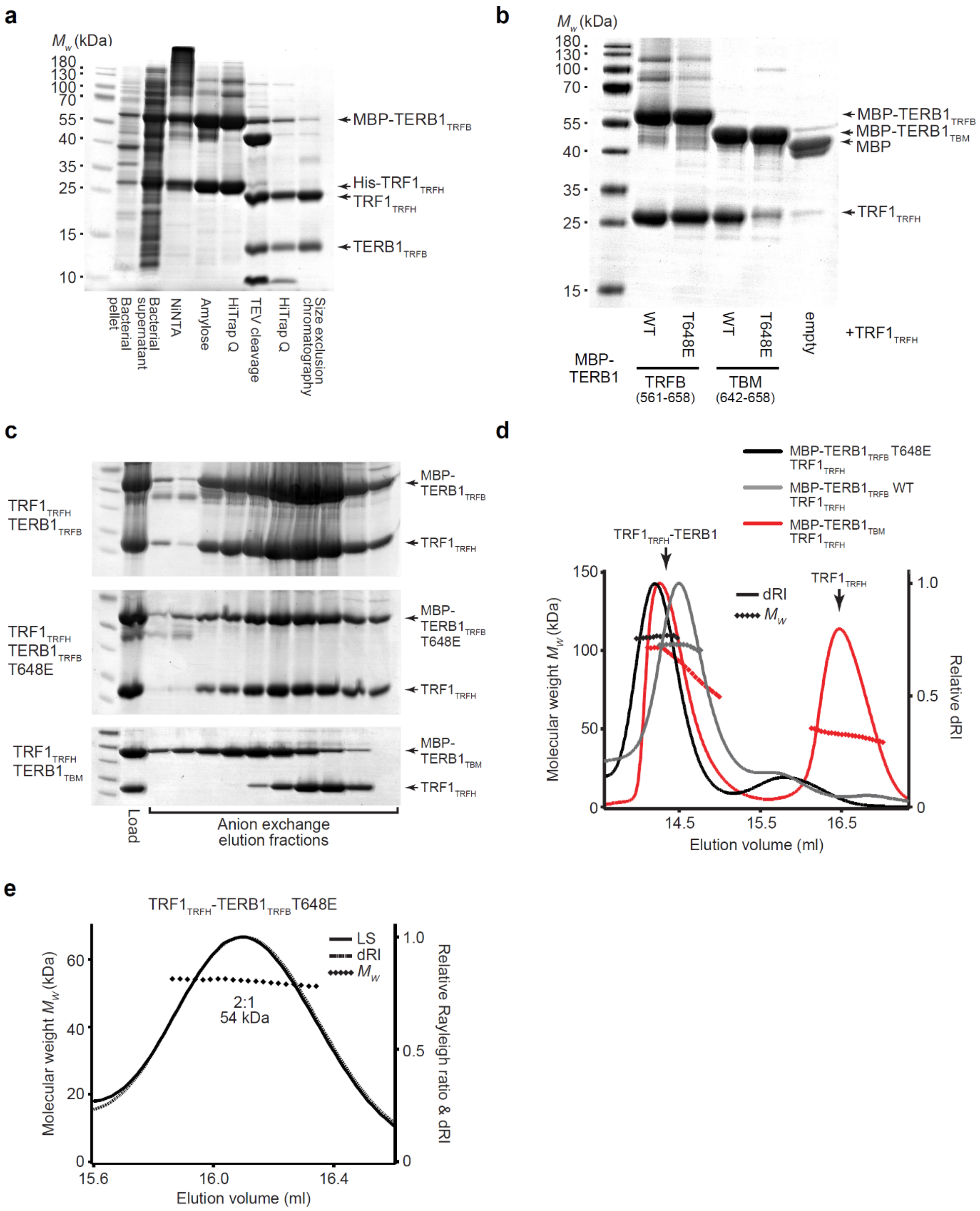

Supplementary Figure 10

Structural stability of TRF1$_{TRFH}$-TERB1 complexes

(**a**) Recombinant co-expression and co-purification of TRF1$_{TRFH}$-TERB1$_{TRFB}$ through Ni-NTA, amylose and anion exchange chromatography, followed by TEV cleavage to remove N-terminal expression tags, with subsequent anion exchange and size exclusion chromatography. (**b**) Amylose pull-down of TRF1$_{TRFH}$ (62-268) with MBP-TERB1$_{TRFB}$ (561-658; wild type and T648E), MBP-TERB1$_{TBM}$ (642-658; wild type and T648E) and free MBP

following recombinant co-expression in bacteria. (**c**) Anion exchange chromatography elution fractions of His-$TRF1_{TRFH}$/MBP-TERB1 complexes. Whilst $TRF1_{TRFH}$ co-purifies with $TERB1_{TRFB}$ and $TERB1_{TRFB}$ T648E, it dissociates from $TERB1_{TBM}$ during anion exchange chromatography. Source data are provided as a Source Data file. (**d**) SEC-MALS analysis of His-$TRF1_{TRFH}$/MBP-TERB1 complexes. His-$TRF1_{TRFH}$/MBP-$TERB1_{TRFB}$ T648E (black) and WT (grey) form stable 2:1 complexes of 109 kDa and 103 kDa, respectively (theoretical 2:1 – 112 kDa), whereas His-$TRF1_{TRFH}$/MBP-$TERB1_{TBM}$ (red) shows partial dissociation from a 99 kDa 2:1 complex (theoretical 2:1 – 103 kDa) to a 46 kDa His-$TRF1_{TRFH}$ dimer (theoretical dimer – 55 kDa). (**e**) SEC-MALS analysis of $TRF1_{TRFH}$-$TERB1_{TRFB}$ T648E demonstrating the formation of a stable 2:1 complex of 54 kDa (theoretical 2:1 – 59 kDa).

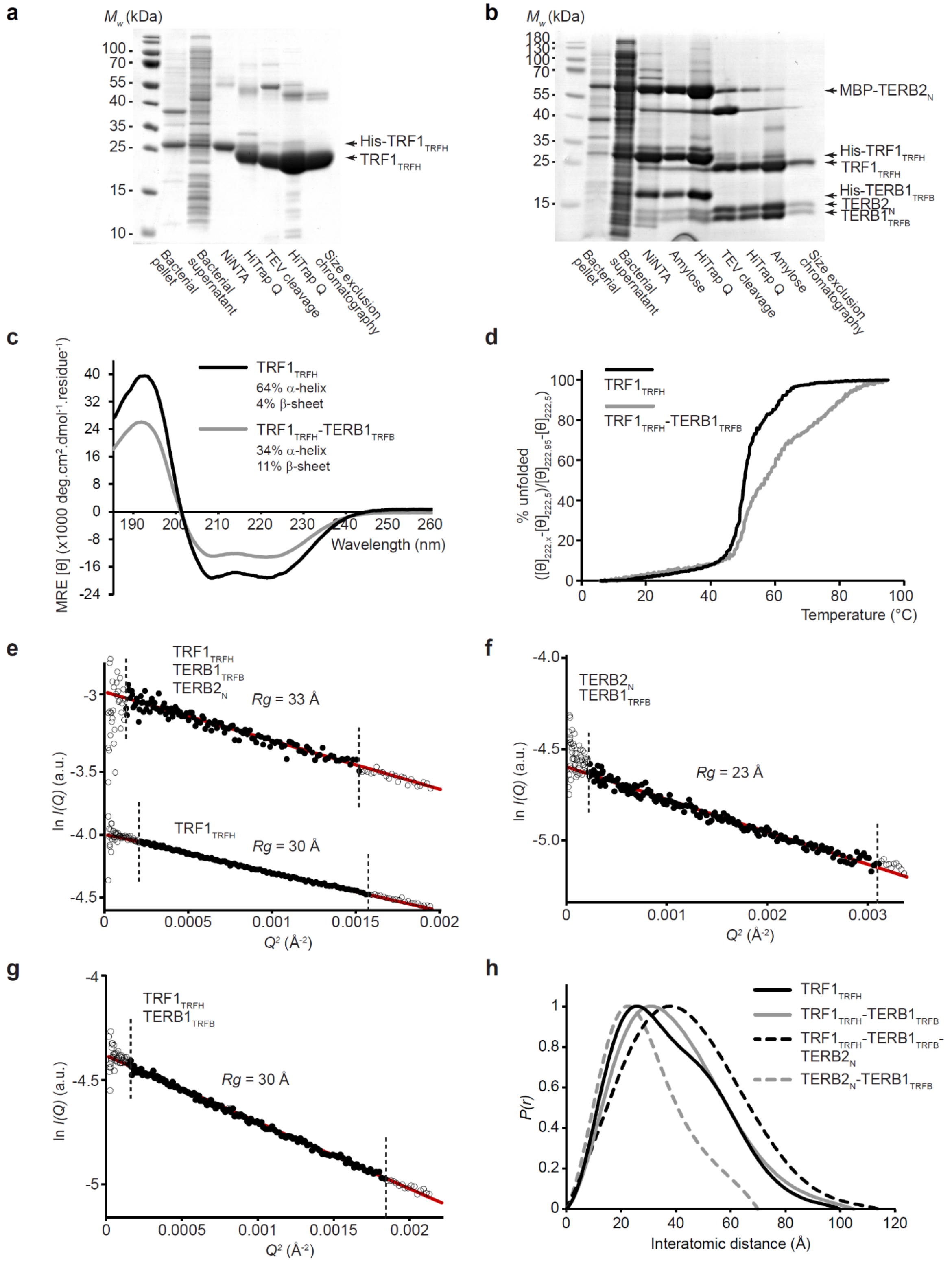

Supplementary Figure 11

Solution structure of $TRF1_{TRFH}$-$TERB1_{TRFB}$-$TERB2_{N}$

(**a**,**b**) Recombinant expression and purification of (**a**) $TRF1_{TRFH}$ (62-268) and (**b**) $TRF1_{TRFH}$-$TERB1_{TRFB}$-$TERB2_{N}$ (62-268, 561-658, 1-119) through Ni-NTA, amylose (as indicated) and anion exchange chromatography, followed

by TEV cleavage to remove N-terminal expression tags, with subsequent anion exchange and size exclusion chromatography. A longer $TERB2_N$ construct of 1-119 was used for size exclusion chromatography and SDS-PAGE to provide molecular weight discrimination from $TERB1_{TRFB}$; other experiments use a shorter 1-107 construct. Source data are provided as a Source Data file. (**c**) Far UV CD spectra and (**d**) CD thermal denaturation of $TRF1_{TRFH}$ (black) and $TRF1_{TRFH}$-$TERB1_{TRFB}$ (grey). (**c**) Secondary structure composition was estimated through deconvolution of spectra with data fitted at normalised rms deviation values of 0.012 and 0.011, respectively. (**d**) Thermal denaturation was recorded as % unfolded based on the helical signal at 222 nm; melting temperatures were estimated at 50°C and 57°C, respectively. (**e**-**h**) SAXS analysis of $TRF1_{TRFH}$, $TRF1_{TRFH}$-$TERB1_{TRFB}$, $TRF1_{TRFH}$-$TERB1_{TRFB}$-$TERB2_N$ and $TERB2_N$-$TERB1_{TRFB}$. (**e**-**g**) Guinier analysis to determine the radius of gyration (*Rg*); the Guinier regions are shown in black, demarcated by vertical dashed lines, with linear fits shown in red (*Q.Rg* values were < 1.3). (**h**) *P(r)* distributions of $TRF1_{TRFH}$ (black, solid), $TRF1_{TRFH}$-$TERB1_{TRFB}$ (grey, solid), $TRF1_{TRFH}$-$TERB1_{TRFB}$-$TERB2_N$ (black, dashed) and $TERB2_N$-$TERB1_{TRFB}$ (grey, dashed), showing maximum dimensions of 100 Å, 105 Å, 114 Å and 70 Å, respectively. Their real space *Rg* values of 30 Å, 31 Å, 34 Å and 23 Å closely match their Guinier analysis *Rg* values of 30 Å, 30 Å, 33 Å and 23 Å, respectively.

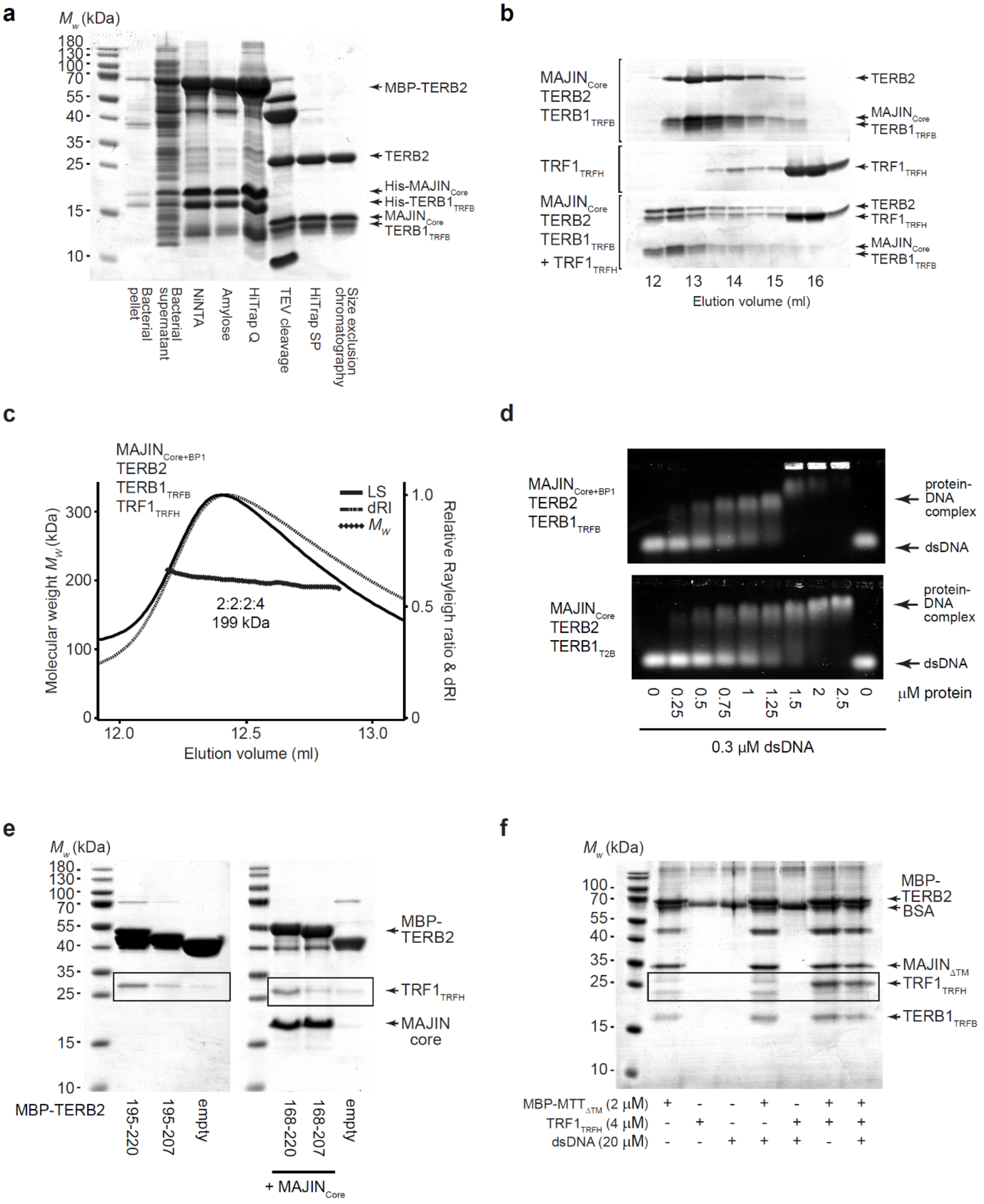

Supplementary Figure 12

$\text{TRF1}_{\text{TRFH}}$- and DNA-binding of $\text{MAJIN}_{\text{Core}}$-TERB2-$\text{TERB1}_{\text{TRFB}}$

(**a**) Recombinant co-expression and co-purification of $\text{MAJIN}_{\text{Core}}$-TERB2-$\text{TERB1}_{\text{TRFB}}$ (1-112, 1-220, 561-658) through Ni-NTA, amylose and anion exchange chromatography, followed by TEV cleavage to remove N-terminal expression tags, with subsequent cation exchange and size exclusion chromatography. (**b**) Size-exclusion chromatography elution profiles of $\text{MAJIN}_{\text{Core}}$-TERB2-$\text{TERB1}_{\text{TRFB}}$, $\text{TRF1}_{\text{TRFH}}$ (62-268), and $\text{MAJIN}_{\text{Core}}$-

TERB2-TERB1$_{TRFB}$ upon incubation with excess TRF1$_{TRFH}$. (**c**) SEC-MALS analysis of MAJIN$_{Core+BP1}$-TERB2-TERB1$_{TRFB}$-TRF1$_{TRFH}$ demonstrating a 2:2:2:4 complex of 199 kDa (theoretical 2:2:2:4 – 206 kDa). (**d**) EMSA analysing the ability of MAJIN$_{Core+BP1}$-TERB2-TERB1$_{TRFB}$ (top) and MAJIN$_{Core}$-TERB2-TERB1$_{T2B}$ (bottom) to interact with linear dsDNA. (**e**) Recombinant co-expression amylose pull-down of His-TRF1$_{TRFH}$ with MBP-TERB2 195-220 and 195-207 (left), complexes between MAJIN$_{Core}$ and MBP-TERB2 168-220 and 168-207 (right), and free MBP. (**f**) Amylose pull-down of TRF1$_{TRFH}$ using MBP-fusion MAJIN$_{\Delta TM}$-TERB2-TERB1$_{TRFB}$ (MBP-MTT$_{\Delta TM}$) with or without pre-incubation with plasmid dsDNA (as indicated). Source data are provided as a Source Data file.

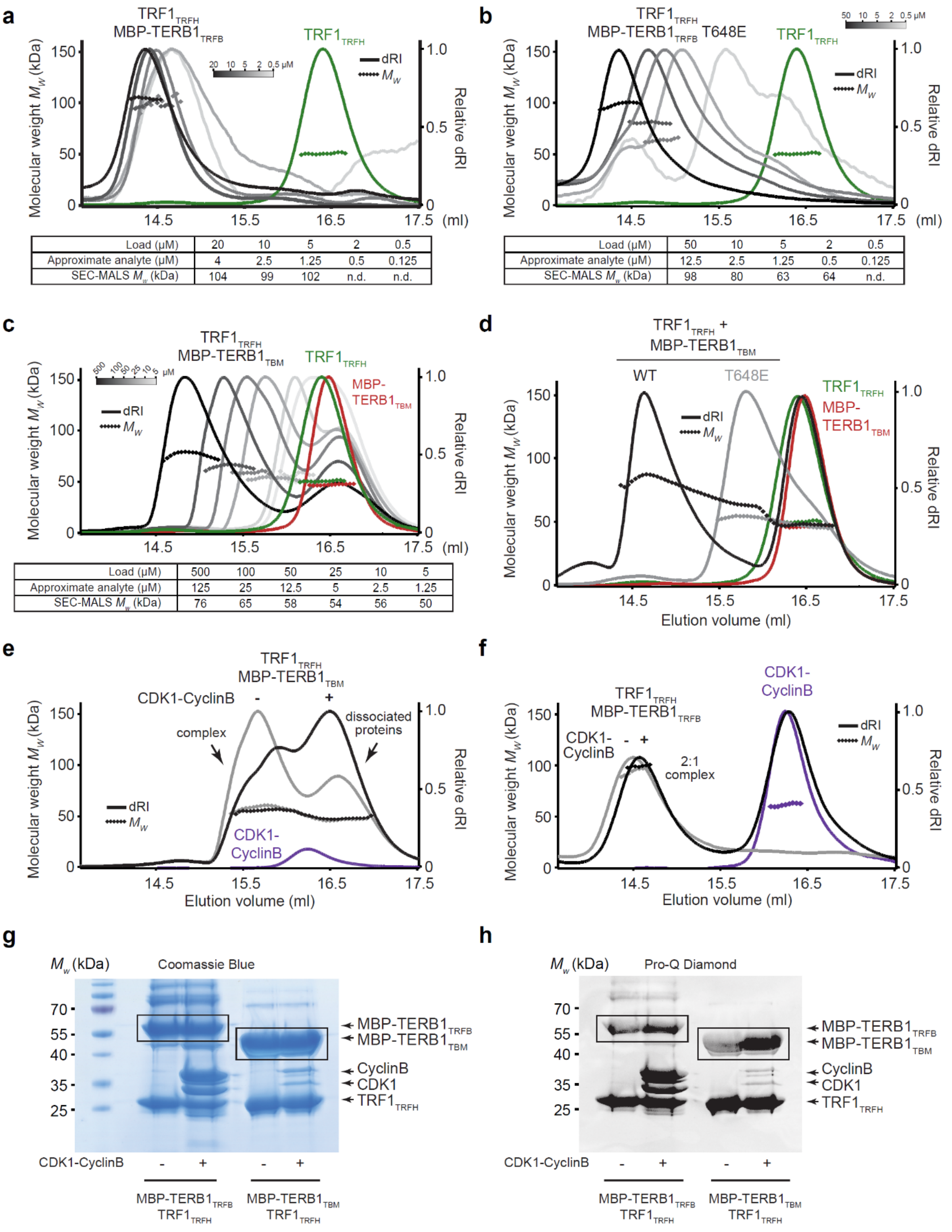

a

Load (μM)	20	10	5	2	0.5
Approximate analyte (μM)	4	2.5	1.25	0.5	0.125
SEC-MALS M_w (kDa)	104	99	102	n.d.	n.d.

b

Load (μM)	50	10	5	2	0.5
Approximate analyte (μM)	12.5	2.5	1.25	0.5	0.125
SEC-MALS M_w (kDa)	98	80	63	64	n.d.

c

Load (μM)	500	100	50	25	10	5
Approximate analyte (μM)	125	25	12.5	5	2.5	1.25
SEC-MALS M_w (kDa)	76	65	58	54	56	50

Supplementary Figure 13

SEC-MALS analysis of TRF1$_{TRFH}$-TERB1 binding

(**a**-**c**) SEC-MALS analysis of His-TRF1$_{TRFH}$ MBP-TERB1 complexes over dilution series. The concentration at which the protein was loaded onto SEC is indicated alongside a conservative estimate (5-fold; experimental data

indicate a dilution of 5-10-fold) of the concentration at which the sample was analysed by MALS, and the determined MALS molecular weight. $TRF1_{TRFH}$ is shown in green for comparison. (**a**) $TRF1_{TRFH}$ and MBP-$TERB1_{TRFB}$ form a clear 2:1 complex by MALS when loaded at 20, 10 and 5 µM (theoretical 2:1 – 111 kDa). Lower concentrations did not produce interpretable MALS data; elution profiles when loaded at 2 and 0.5 µM suggest that complex formation is largely retained but with signs of some dissociation. (**b**) $TRF1_{TRFH}$ and MBP-$TERB1_{TRFB}$ T648E form a clear 2:1 complex by MALS when loaded at 50 µM (theoretical 2:1 – 111 kDa). Lower concentrations show progressively reducing molecular weight, indicating some dissociation of the complex; free $TRF1_{TRFH}$ is clearly observed when loaded at 0.5 µM. These traces suggest an approximately 10-fold reduction in affinity with respect to the wild type complex. (**c**) $TRF1_{TRFH}$ and MBP-$TERB1_{TBM}$ undergoes only partial complex formation when loaded at 500 µM, with the molecular weight suggesting a mixture between a 2:1 complex (102 kDa) and free $TRF1_{TRFH}$ dimer (55 kDa). Further dilutions show a progressive reduction in molecular weight, with complete dissociation observed when loaded at 5 µM. These traces suggest a reduction in affinity by more than 100-fold relative to wild type $TERB1_{TRFB}$, and more than 10-fold relative to $TERB1_{TRFB}$ T648E. (**d**) SEC-MALS analysis of MBP- $TERB1_{TBM}$ wild type and T648E (loaded at 600 µM) following incubation with a 2-fold molar excess of $TRF1_{TRFH}$, indicating only partial complex formation of wild type as the molecular weight is less than the theoretical 2:1 mass of 102 kDa. (**e**-**h**) In vitro phosphorylation of His-$TRF1_{TRFH}$ MBP-TERB1 by CDK-CyclinB. (**e**,**f**) SEC-MALS analysis of (**e**) $TRF1_{TRFH}$ MBP-$TERB1_{TBM}$ (130 µM) and (**f**) $TRF1_{TRFH}$ MBP-$TERB1_{TRFB}$ (9 µM) upon treatment with 9 µM CDK1-CyclinB (isolated sample shown in purple for comparison). Whilst sub-stoichiometric quantities of CDK1-CyclinB are sufficient to induce dissociation of $TRF1_{TRFH}$ MBP-$TERB1_{TBM}$, equimolar concentrations fail to dissociate $TRF1_{TRFH}$ MBP-$TERB1_{TRFB}$. (**g**,**h**) SDS-PAGE analysis of phosphorylated protein samples in which gels were stained with (**g**) Coomassie blue (total protein) and (**h**) Pro-Q Diamond (preferential staining of phosphorylated proteins). The enhanced staining of MBP-$TERB1_{TBM}$ upon CDK1-CyclinB treatment indicates successful phosphorylation; a lower level of enhancement of MBP-$TERB1_{TRFB}$ staining indicates a reduced susceptibility to phosphorylation despite its treatment with a 10-fold higher molar ratio of CDK1-CyclinB. Source data are provided as a Source Data file.

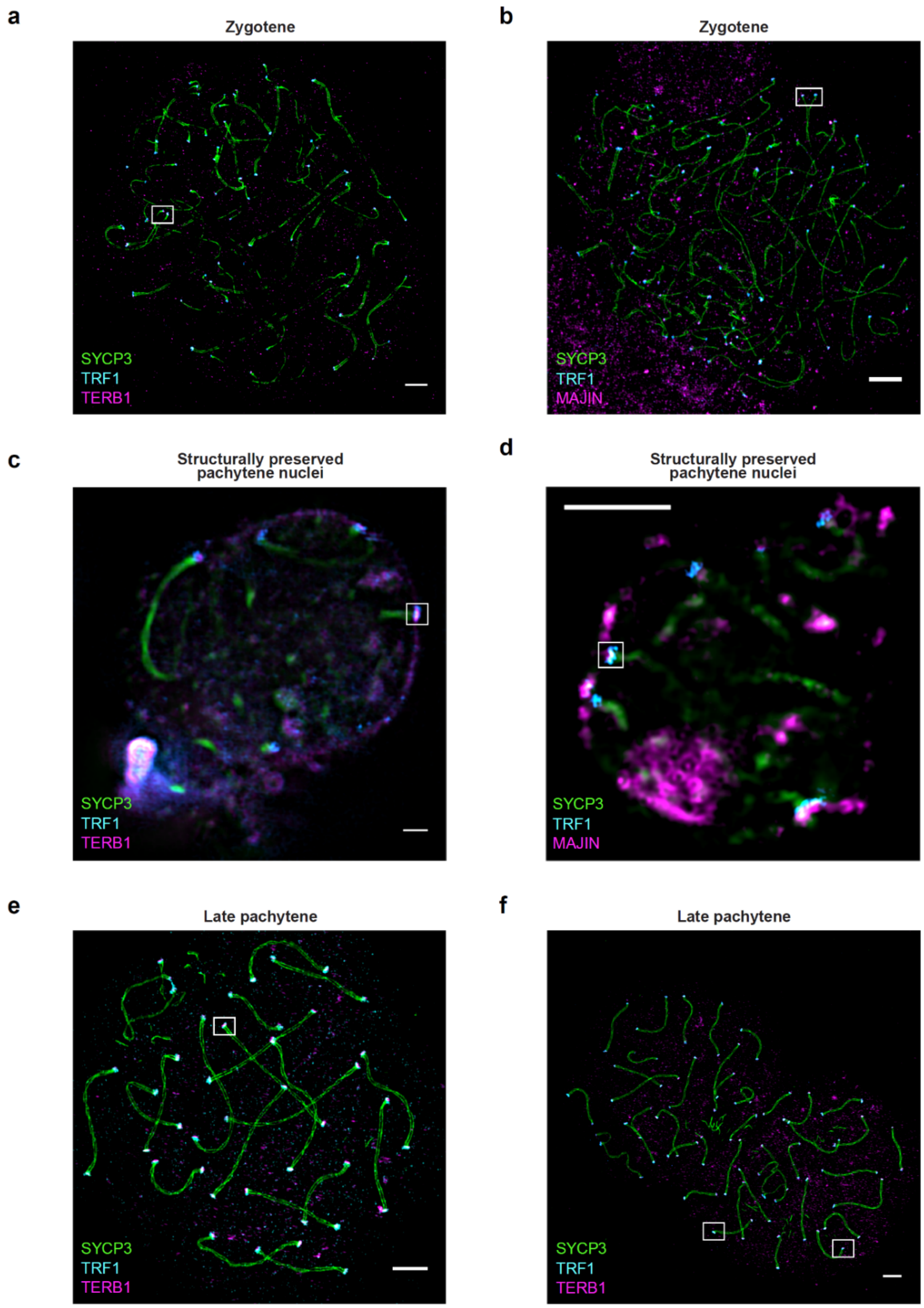

Supplementary Figure 14

Wide field SIM images of TRF1 and telomeric DNA

Structured illumination microscopy of (**a**,**b**) mouse zygotene spermatocyte chromosome spreads, (**c**,**d**) structurally preserved mouse pachytene nuclei and (**e**,**f**) mouse pachytene spermatocyte chromosome spreads stained with anti-SYCP3 (green), anti-TRF1 (cyan) and (**a**,**c**,**e**,**f**) anti-TERB1 (magenta) or (**b**,**d**) anti-MAJIN (magenta). Scale bars, 3 µm. White squares represent the telomere ends used for display and analysis in (**a**,**b**) Fig. 8a, (**c**,**d**) Fig. 8b and (**e**,**f**) Fig. 8c.

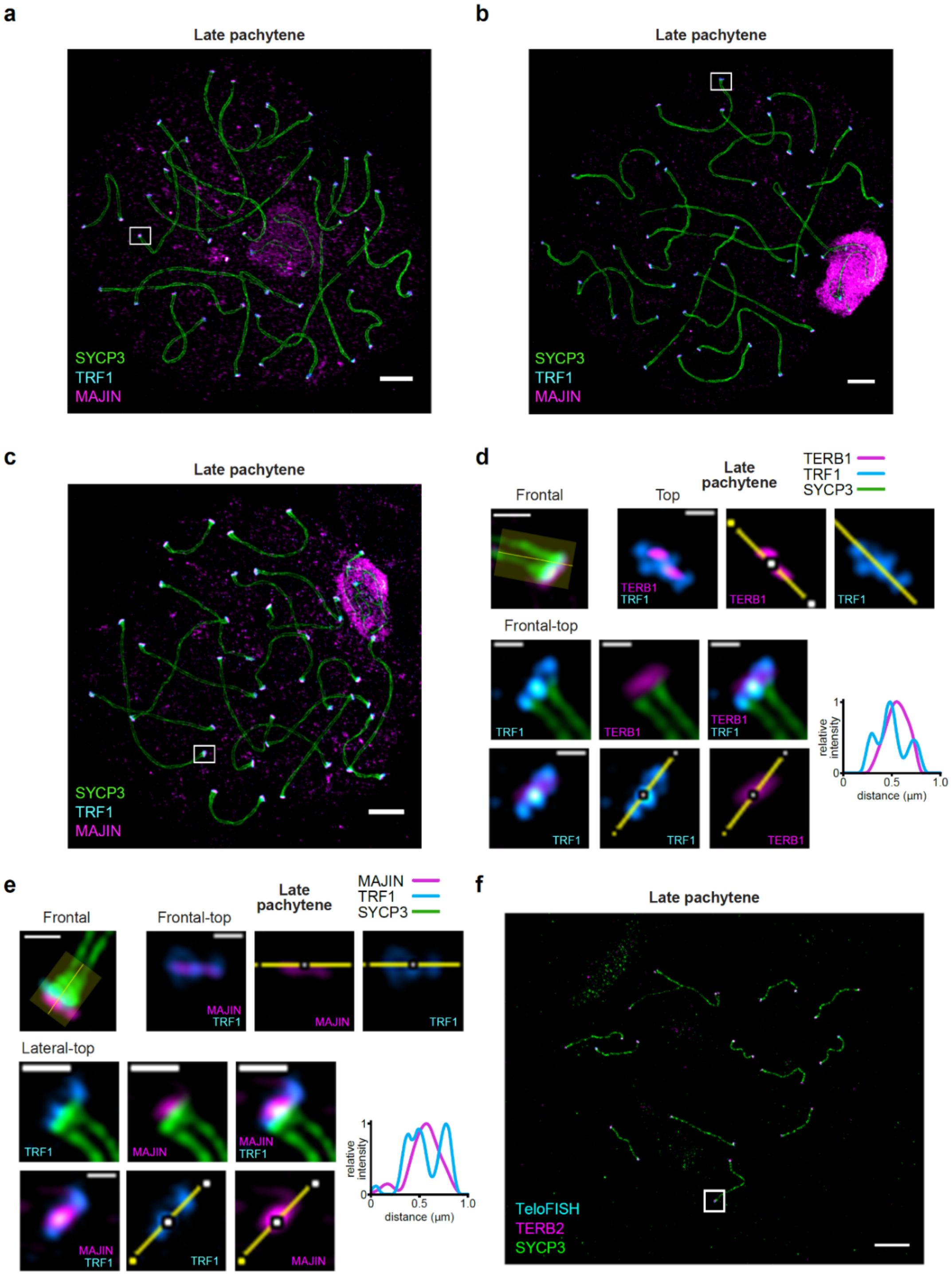

Supplementary Figure 15

Wide field SIM images and SIM analyses of TRF1 and telomeric DNA

Structured illumination microscopy of mouse pachytene spermatocyte chromosome spreads stained with anti-SYCP3 (green), (**a**-**e**) anti-TRF1 (cyan) and (**a**-**c**,**e**) anti-MAJIN (magenta) or (**d**) anti-TERB1 (magenta), or (**f**) anti-TERB2 (magenta) in combination with telomere fluorescence *in situ* hybridisation (cyan; TeloFISH). Scale bars, (**a**-**c**,**f**) 3 µm and (**d**,**e**) 0.3 µm. (**a**-**c**,**f**) White squares represent the telomere ends used for display and analysis in (**a**-**c**) Fig. 8d and (**f**) Fig. 8e. (**d**,**e**) Analyses relating to plots shown in Fig. 8c-d.

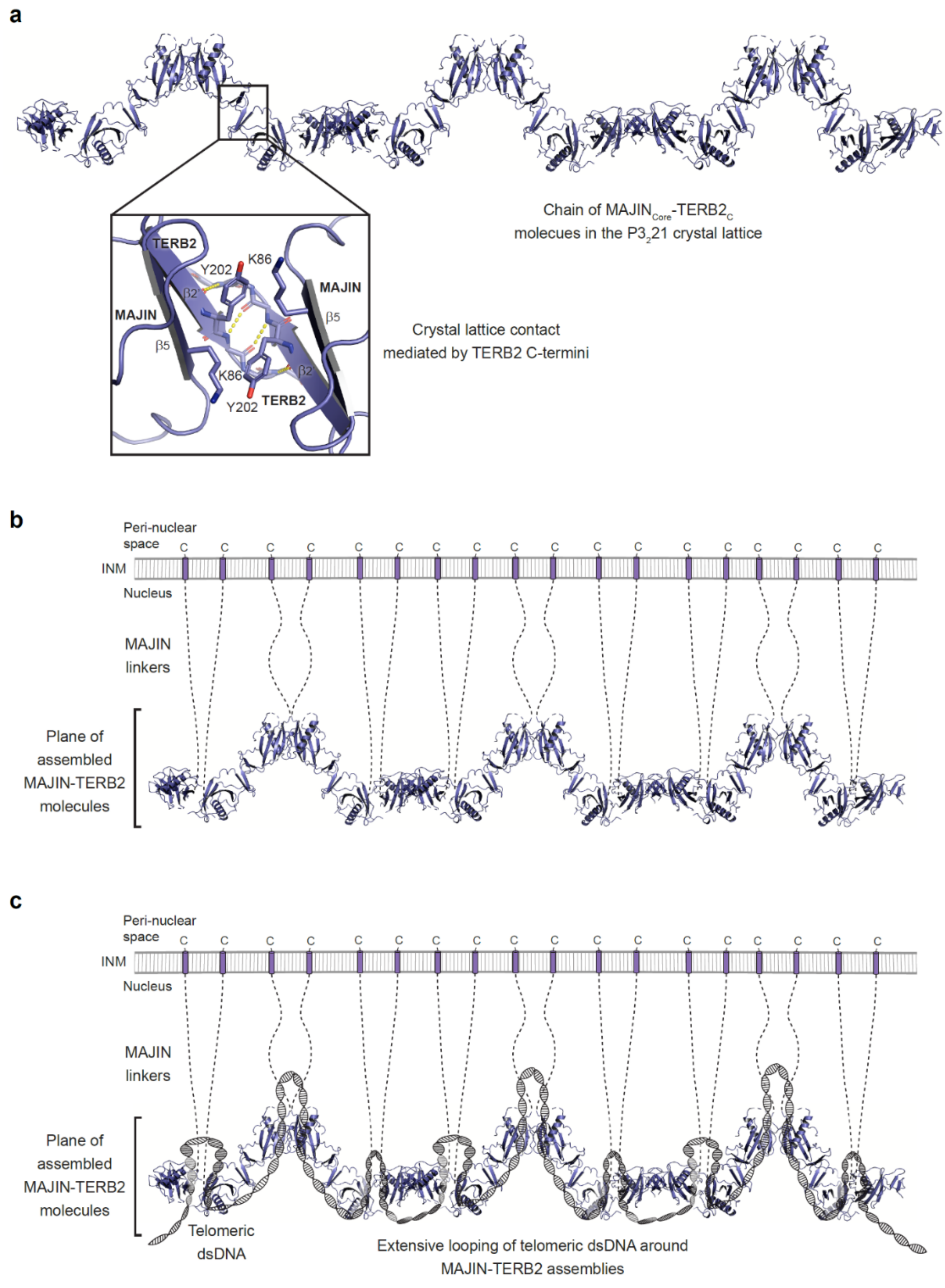

Supplementary Figure 16

Higher order assembly of MAJIN$_{Core}$-TERB2$_C$

(**a**) Linear assembly of MAJIN-TERB2 complexes within the MAJIN$_{Core}$-TERB2$_C$ P3$_2$21 crystal lattice mediated by the MAJIN dimerization interface and interactions between TERB2 C-termini. TERB2 C-terminal contacts are

formed of an anti-parallel β-sheet interaction with hydrophobic packing of residues Y202 (TERB2) and K86 (MAJIN). (**b**) If the crystal lattice is replicated in the cell it may produce a plane of assembled MAJIN-TERB2 complexes oriented vertically and horizontally such that the C-terminal linkers provide a substantial separation to their inner nuclear membrane insertion. (**c**) Speculative model of how telomeric DNA may bind to the aforementioned MAJIN-TERB2 complexes. Telomeric DNA may be looped around MAJIN-TERB2 complexes, which are themselves arranged in higher order assemblies, thus creating extensive interaction sites within telomere end attachment plates.

Supplementary Table 1
Summary of SEC-SAXS data

	$MAJIN_{Core}$<br>$TERB2_{C}$	$MAJIN_{Core\text{-}Tr}$<br>$TERB2_{C\text{-}Tr}$	$MAJIN_{\Delta TM}$<br>$TERB2_{C\text{-}Tr}$	$MAJIN_{Core}$<br>TERB2<br>$TERB1_{T2B}$	$TERB2_{N}$<br>$TERB1_{T2B}$	$TRF1_{TRFH}$<br>$TERB1_{TRFB}$<br>$TERB2_{N}$	$TRF1_{TRFH}$	$TRF1_{TRFH}$<br>$TERB1_{TRFB}$	$TERB2_{N}$<br>$TERB1_{TRFB}$
**Data-collection parameters**									
Beamline	Diamond<br>B21	Diamond<br>B21	Diamond<br>B21	Diamond<br>B21	Diamond<br>B21	Diamond<br>B21	Diamond<br>B21	Diamond<br>B21	Diamond<br>B21
Beam geometry (mm)	1x1	1x1	1x1	1x1	1x1	1x1	1x1	1x1	1x1
Capillary diameter (mm)	1.5	1.5	1.5	1.5	1.5	1.5	1.5	1.5	1.5
Beam strength (photons s^{-1})	$3x10^{12}$	$3x10^{12}$	$3x10^{12}$	$3x10^{12}$	$3x10^{12}$	$3x10^{12}$	$3x10^{12}$	$3x10^{12}$	$3x10^{12}$
Flow rate (ml min^{-1})	0.5	0.5	0.5	0.5	0.5	0.5	0.5	0.5	0.5
Q range ($Å^{-1}$)	0.0037 –<br>0.37	0.0037 –<br>0.37	0.0035 –<br>0.37	0.0035 –<br>0.37	0.0035 –<br>0.37	0.0035 –<br>0.37	0.0037 –<br>0.37	0.0035 –<br>0.37	0.0035 –<br>0.37
Exposure time (s)	0.225	0.225	0.225	0.225	0.225	0.225	0.225	0.225	0.225
Temperature (K)	298	298	298	298	298	298	298	298	298
**Structure parameters**									
I(0) (cm^{-1}) [from *P(r)*]	0.080<br>± $1.7x10^{-4}$	0.065<br>± $6.6x10^{-5}$	0.15<br>± $2.7x10^{-4}$	0.053<br>± $2.8x10^{-4}$	0.059<br>± $4.1x10^{-5}$	0.017<br>± $9.8x10^{-5}$	0.11<br>± $1.3x10^{-4}$	0.037<br>± $5.5x10^{-5}$	0.0095<br>± $2.0x10^{-5}$
Rg (Å) [from *P(r)*]	32.08<br>± 0.095	24.47<br>± 0.026	39.43<br>± 0.15	54.00<br>± 0.49	18.66<br>± 0.021	34.13<br>± 0.21	29.65<br>± 0.040	30.94<br>± 0.054	22.52<br>± 0.065
I(0) (cm^{-1}) [from Guinier]	0.079<br>± $1.7x10^{-4}$	0.065<br>± $8.3x10^{-5}$	0.14<br>± $2.7x10^{-4}$	0.051<br>± $2.3x10^{-4}$	0.059<br>± $4.6x10^{-5}$	0.017<br>± $1.1x10^{-4}$	0.11<br>± $1.4x10^{-4}$	0.037<br>± $5.5x10^{-5}$	0.0097<br>± $2.8x10^{-5}$
Rg (Å) [from Guinier]	30.25<br>± 1.76	24.21<br>± 4.14	37.09<br>± 0.11	48.63<br>± 0.34	18.50<br>± 0.02	33.40<br>± 0.33	30.23<br>± 0.06	30.26<br>± 0.07	23.07<br>± 2.18
D_{max} (Å)	120	80	155	220	65	114	100	105	70
Porod volume estimate ($Å^3$)	83883	62981	124522	170974	30461	145259	79647	103062	45023
Molecular mass M_r [from Porod volume] (kDa)*	49.3	37.0	73.2	100.6	17.9	85.4	46.9	60.6	26.5
Calculated monomer M_r from sequence (kDa)	13.6<br>6.3	12.7<br>4.7	26.7<br>4.7	13.6<br>26.2<br>7.0	12.8<br>6.8	23.7<br>11.6<br>12.8	23.7	23.7<br>11.6	12.8<br>11.6
Oligomer determined by SEC-MALS	2:2	2:2	2:2	2:2:2	1:1	2:1:1	Dimer	2:1	1:1
Calculated oligomer M_r from sequence (kDa)	39.7	34.9	62.9	93.6	19.6	71.8	47.3	58.9	24.4
**Software employed**									
Data processing	ScÅtter	ScÅtter	ScÅtter	ScÅtter	ScÅtter	ScÅtter	ScÅtter	ScÅtter	ScÅtter
Ab initio analysis	N/A	DAMMIF	N/A	N/A	DAMMIF	MONSA	MONSA	MONSA	MONSA
Validation and averaging	N/A	DAMAVER	N/A	N/A	DAMAVER	N/A	N/A	N/A	N/A
Rigid-body modelling	N/A	N/A	CORAL	CORAL	N/A	N/A	N/A	N/A	N/A
Computation of model intensities	N/A	CRYSOL	CRYSOL	CRYSOL	N/A	CRYSOL	CRYSOL	CRYSOL	CRYSOL
Three-dimensional graphics representation	PyMOL	PyMOL	PyMOL	PyMOL	PyMOL	PyMOL	PyMOL	PyMOL	PyMOL

*Molecular mass M_r was calculated by dividing the Porod volume by 1.7.

Supplementary Table 2
Primer sequences used in this study

Plasmid	Template sequence	Amino-acid residue start/end	Forward/reverse primer	Primer sequence
pHAT4/pMAT11	TRF1	1	Forward	TTCCAGGGTTCCATGGCGGAGGATGTTTCCTC
pHAT4/pMAT11	TRF1	62	Forward	TTCCAGGGTTCCATGGAGGACGCGGGCCTGGTGGCC
pHAT4/pMAT11	TRF1	439	Reverse	AATTCGATATCCATGGTTATTAGTCTTCGCTGTCTGAGGAAATC
pHAT4/pMAT11	TRF1	268	Reverse	AATTCGATATCCATGGTTATTACCTTTTGCTTTCTACTACTTTTG
pRSF-Duet1 (2)	TRF1	268	Reverse	TTGAGATCTGCCATATGTTATTACCTTTTGCTTTCTACTACTTTTG
pHAT4/pMAT11	TERB1	561	Forward	TTCCAGGGTTCCATGACCGATCCGTTTACACTGTG
pHAT4/pMAT11	TERB1	658	Reverse	AATTCGATATCCATGGTTATTATTCATTGCTCAGACGCTGACG
pHAT4/pMAT11	TERB1	642	Forward	TTCCAGGGTTCCATGAACAAAAAGATTCTGCTGAC
pHAT4/pMAT11	TERB1	585	Forward	TTCCAGGGTTCCATGAGCGAAATGCTGACCTATCGTTG
pHAT4/pMAT11	TERB1	642	Reverse	AATTCGATATCCATGGTTATTAGTTGCAGATCAGACTTTTACG
pHAT4/pMAT11	TERB1 (T648E)	658	Reverse	AATTCGATATCCATGGTTATTATTCATTGCTCAGACGCTGACGACGACGCGGT TCCAGCAGAATCTTTTTG
pHAT4/pMAT11	TERB1 (T648E)	642	Forward	AATTCGATATCCATGGTTATTATTCATTGCTCAGACGCTGACGACGACGCGGT TCCAGCAGAATCTTTTTG
pHAT4/pMAT11	TERB2	1	Forward	TTCCAGGGTTCCATGACCGGCAGCACAGGTTCTACCGGCAGCTTTCAGGGTC AGCGTGGTTGG
pHAT4/pMAT11	TERB2	147	Forward	TTCCAGGGTTCCATGGAAAAACACTTTATTCGTAC
pHAT4/pMAT11	TERB2	168	Forward	TTCCAGGGTTCCCCGGTGAATAATATGGTTACC
pHAT4/pMAT11	TERB2	195	Forward	TTCCAGGGTTCCATGGGTACAAGCGGTTATCTG
pHAT4/pMAT11	TERB2	196	Reverse	AATTCGATATCCATGGTTATTATGTACCCGGAATAAAGTC
pHAT4/pMAT11	TERB2	207	Reverse	AATTCGATATCCATGGTTATTATTCGTTCTGAACATGATATGC
pHAT4/pMAT11	TERB2	220	Reverse	AATTCGATATCCATGGTTATTATTTGCGCTTCAGTTTGTTTTTG
pHAT4/pMAT11	TERB2	119	Reverse	AATTCGATATCCATGGTTATTATTCATCGTGTTTTTCGATCAG
pHAT4/pMAT11	TERB2	107	Reverse	AATTCGATATCCATGGTTATTATTCCCAGATAAAGCTGCC
pRSF-Duet1 (1)	TERB2	220	Reverse	TGGCTGCTGCCCATGGTTATTATTTGCGCTTCAGTTTGTTTTTG
pRSF-Duet1 (1)	TERB2	119	Reverse	TGGCTGCTGCCCATGGTTATTATTCATCGTGTTTTTCGATCAG
pRSF-Duet1 (1)	TERB2	107	Reverse	TGGCTGCTGCCCATGGTTATTATTCCCAGATAAAGCTGCC
pRSF-Duet1 (2)	TERB2	119	Reverse	TTGAGATCTGCCATATGTTATTATTCATCGTGTTTTTCGATCAG
pRSF-Duet1 (2)	TERB2	220	Reverse	TTGAGATCTGCCATATGTTATTATTTGCGCTTCAGTTTGTTTTTG
pHAT4/pMAT11	TERB2	207	Reverse	AATTCGATATCCATGGTTATTATTCGTTCTGAACATGATATGC
pHAT4/pMAT11	MAJIN	1	Forward	TTCCAGGGTTCCATGAGTTTAAAACCCTTTACCTAC
pHAT4/pMAT11	MAJIN	233	Reverse	AATTCGATATCCATGGTTATTACTCGTCCATGTGTTTTCGTTTC
pHAT4/pMAT11	MAJIN	147	Reverse	AATTCGATATCCATGGTTATTA CTCGTCCATGTGTTTTCGTTTCCTCATAACGGCACCAACTGCTTTTTTTTCAACC GGAACCAGACCCAGCGGACTATC
pHAT4/pMAT11	MAJIN	112	Reverse	AATTCGATATCCATGGTTATTATTCGTGGAACCATTTCATTTC
pHAT4/pMAT11	MAJIN	106	Reverse	TGGCTGCTGCCCATGGTTATTACTCCACATATAGAGTAAA
pRSF-Duet1 (1)	MAJIN	112	Reverse	TGGCTGCTGCCCATGGTTATTATTCGTGGAACCATTTCATTTC
pRSF-Duet1 (2)	MAJIN	112	Reverse	TTGAGATCTGCCATATGTTATTATTCATGGAACCATTTCAT
pRSF-Duet1 (1)	MAJIN	233	Reverse	TGGCTGCTGCCCATGGTTATTACTCGTCCATGTGTTTTCGTTTC
pRSF-Duet1 (2)	MAJIN	233	Reverse	TTGAGATCTGCCATATGTTATTACTCGTCCATGTGTTTTCGTTTC
NA (mutagenesis)	MAJIN	F73E Y75E	Forward	GAACATTTTATTGTGGAACCGGAAAAAAGCAAATGGGAAC
NA (mutagenesis)	MAJIN	F73E Y75E	Reverse	GTTCCCATTTGCTTTTTTCCGGTTCCACAATAAAATGTTC
NA (mutagenesis)	MAJIN	Basic surface mutant	Forward	GGACCCAATGTGTATATGTTCGAAATTGAATACGGCGATAGCATCGAAGGTG AAGAAATTGAAAAC
NA (mutagenesis)	MAJIN	Basic surface mutant	Reverse	GTTTTCAATTTCTTCACCTTCGATGCTATCGCCGTATTCAATTTCGAACATATAC ACATTGGGTCC
NA (mutagenesis)	MAJIN	R81D	Forward	GCAAATGGGAGGATGTTTCCCACCTGAAATTC
NA (mutagenesis)	MAJIN	R81D	Reverse	GAATTTCAGGTGGGAAACATCCTCCCATTTGC
pRSF-Duet1 (2)	His-tag	1	Forward	AAGGAGATATACATATGAACACCATTCATCACCATC
pRSF-Duet1 (1)	MBP-tag	1	Forward	AGGAGATATACCATGGGCAGCAGCATGAAAATCGAAGAAGGTAAACT
pRSF-Duet1 (1)	His-tag	1	Forward	AGGAGATATACCATGGGCAACACCATTCATCACCATC

Supplementary Table 3
DNA substrates used in this study

DNA Substrate	Used for display EMSA or K_D determination	5'-label	Sequence
Random dsDNA (75 bp)	EMSA		GCGAACACCCTGCATCGTCCGACCGGCTCTACAGGTTCCACCGGTTCTACGGGC TCCGGCCTGTATTTCTCTTCC
Random dsDNA (57 bp)	EMSA		GCGAACACCCTGCATCGTCCGACCGGCTCTACAGGTTCCGGCCTGTATTTCTCTT CC
Telomeric dsDNA (hairpin)	EMSA		TTAGGGTTAGGGTTAGGGTTAGGGTTAGGGTTAGGGTTAGGGTTAGGGTTAGG GCGACGACCCTAACCCTAACCCTAACCCTAACCCTAACCCTAACCCTAACCCTAA CCCTAA
Random ssDNA	EMSA		AATTCTCATTTTACTTACCGGACGCTATTAGCAGTGGCAGATTGTACTGAGAGTG CACCATATGCGGTGTGAAATACCGCACAGATGCGT
Poly (dT) ssDNA	EMSA		TTTTTTTTTTTTTTTTTTTTTTTTTTTTTTTTTTTTTTTTTTTTTTTTTTTTTTTTTTTTTT TTTTTTTTTTTTTTTTTTTTTTTTTTTTTTTTTTTTTT
Random dsDNA	K_D determination	5' 6-FAM	TTCCAGGGTTCCATGGAGATAAAGGTCAATAAATTAGAGTTAGAACTAGAAAGT GCCAAACAGAAATTTGGAGAAATCACAGACACCTATCAGAAAGAAATTGAGGA CAAAAAGATATCAGAATAATAACCATGGATATCGAATT
Telomeric dsDNA (hairpin)	K_D determination		TTAGGGTTAGGGTTAGGGTTAGGGTTAGGGTTAGGGTTAGGGTTAGGGTTAGG GCGACGACCCTAACCCTAACCCTAACCCTAACCCTAACCCTAACCCTAACCCTAA CCCTAA
Random ssDNA	K_D determination	5' 6-FAM	AATTCTCATTTTACTTACCGGACGCTATTAGCAGTGGCAGATTGTACTGAGAGTG CACCATATGCGGTGTGAAATACCGCACAGATGCGT
Poly (dT) ssDNA	K_D determination	5' 6-FAM	TTTTTTTTTTTTTTTTTTTTTTTTTTTTTTTTTTTTTTTTTTTTTTTTTTTTTTTTTTTTTT TTTTTTTTTTTTTTTTTTTTTTTTTTTTTTTTTTTTTT
