# Supplementary material for: Structural basis of meiotic telomere attachment to the nuclear envelope by MAJIN-TERB2-TERB1
Source: Nat Commun. 2018 Dec 17;9:5355. doi: 10.1038/s41467-018-07794-7 (PMC6297230; doi:10.1038/s41467-018-07794-7)
Supplement: Supplementary file 3 — Reporting Summary [file 41467_2018_7794_MOESM3_ESM.pdf]

nature research

Corresponding author(s): Dr. Owen Richard Davies

Life Sciences Reporting Summary

Nature Research wishes to improve the reproducibility of the work that we publish. This form is intended for publication with all accepted life science papers and provides structure for consistency and transparency in reporting. Every life science submission will use this form; some list items might not apply to an individual manuscript, but all fields must be completed for clarity.

For further information on the points included in this form, see Reporting Life Sciences Research. For further information on Nature Research policies, including our data availability policy, see Authors & Referees and the Editorial Policy Checklist.

Please do not complete any field with "not applicable" or n/a. Refer to the help text for what text to use if an item is not relevant to your study. For final submission: please carefully check your responses for accuracy; you will not be able to make changes later.

Experimental design

1. Sample size

Describe how sample size was determined.

The volume and concentration of protein samples analysed in biochemical and biophysical analyses were selected to provide sufficient signal-to-noise for accurate data analysis. Protein concentration in crystallisation experiments was as required for crystal growth.

2. Data exclusions

Describe any data exclusions.

All gel images presented in the main text are included as uncropped images in Supplementary Figures and in the Source Data file.

3. Replication

Describe the measures taken to verify the reproducibility of the experimental findings.

All biochemical and biophysical experiments were repeated at least three times with separately prepared recombinant protein material. Quantification of each individual electrophoretic mobility shift assay was performed in triplicate, and the mean value of each averaged with that of a further two repeat assays. The resultant values are plotted with error bars representing standard error.

4. Randomization

Describe how samples/organisms/participants were allocated into experimental groups.

Protein samples were not grouped into experimental groups as this is not required for the study.

5. Blinding

Describe whether the investigators were blinded to group allocation during data collection and/or analysis.

Investigators were not blinded as this is not applicable to our study.

Note: all in vivo studies must report how sample size was determined and whether blinding and randomization were used.

6. Statistical parameters

For all figures and tables that use statistical methods, confirm that the following items are present in relevant figure legends (or in the Methods section if additional space is needed).

n/a	Confirmed	
☐	☒	The exact sample size (*n*) for each experimental group/condition, given as a discrete number and unit of measurement (animals, litters, cultures, etc.)
☒	☐	A description of how samples were collected, noting whether measurements were taken from distinct samples or whether the same sample was measured repeatedly
☐	☒	A statement indicating how many times each experiment was replicated
☒	☐	The statistical test(s) used and whether they are one- or two-sided<br>*Only common tests should be described solely by name; describe more complex techniques in the Methods section.*
☒	☐	A description of any assumptions or corrections, such as an adjustment for multiple comparisons
☒	☐	Test values indicating whether an effect is present<br>*Provide confidence intervals or give results of significance tests (e.g. P values) as exact values whenever appropriate and with effect sizes noted.*
☒	☐	A clear description of statistics including central tendency (e.g. median, mean) and variation (e.g. standard deviation, interquartile range)
☐	☒	Clearly defined error bars in all relevant figure captions (with explicit mention of central tendency and variation)

See the web collection on statistics for biologists for further resources and guidance.

Software

Policy information about availability of computer code

7. Software

Describe the software used to analyze the data in this study.

ASTRA® 6 software - MALS data analysis
ScÅtter 3.0 - SAXS data analysis
ATSAS software suite (PRIMUS, DAMMIF, SUPCOMB, CORAL, MONSA) - SAXS data analysis, ab initio modelling, rigid body and linker fitting
www.bayesapp.org - SAXS data analysis
XDS - X-ray diffraction data processing
XSCALE - X-ray diffraction data scaling
AutoPROC - X-ray diffraction data processing
CCP4 Aimless - X-ray diffraction data merging
Phaser - Crystallographic molecular replacement
PHENIX - Crystallographic experimental phasing, model building and refinement
Coot - Crystallographic model visualisation and manual building
Consurf - Protein sequence conservation analysis
JNet - Protein secondary structure prediction
PISA - Protein structure assembly analysis
PyMOL version 1.3 - Molecular graphics software
Dichroweb CDSSTR algorithm - CD data deconvolution
Zen 2012 SP1 (Black edition) Elyra release version 8.1 Carl Zeiss Microscopy GmbH 1997-2013
ImageJ 1.52e Wayne Rasband National Institutes of Health, USA
GraphPad Prism 8.0.0.

For manuscripts utilizing custom algorithms or software that are central to the paper but not yet described in the published literature, software must be made available to editors and reviewers upon request. We strongly encourage code deposition in a community repository (e.g. GitHub). *Nature Methods* guidance for providing algorithms and software for publication provides further information on this topic.

Materials and reagents

Policy information about availability of materials

8. Materials availability

Indicate whether there are restrictions on availability of unique materials or if these materials are only available for distribution by a third party.

There are no restrictions on any materials used in this study.

9. Antibodies

Describe the antibodies used and how they were validated for use in the system under study (i.e. assay and species).	Primary antibodies used in this study were as follows: rabbit antibodies against mouse TRF1 (Alpha Diagnostic; TRF12-A), TERB2 (Shibuya et al., 2015); mouse against mouse SYCP3 (Abcam; ab 97672), guinea pig against C-terminus (1095-1585 aa) rat SYCP2 (Seqlab; Götingen, Germany), against 13-aa from 18-30 aa N-terminus mice MAJIN (GenBank accession number: BAT24489) (Seqlab; Götingen, Germany), against 15-aa from C-terminus 525-540 aa mice TERB1 (GenBank accession number: NP_851289) (Seqlab; Götingen, Germany) and against and against 103-aa from the C-terminal of mice TERB1.<br>The antibodies were validated in several single, double a triple indirect immunofluorescence assays. The antibodies are specific for telomeres of meiotic cells. As expected, they do not recognize telomeres of somatic cells

10. Eukaryotic cell lines

a. State the source of each eukaryotic cell line used.	No eukaryotic cell lines were used.
b. Describe the method of cell line authentication used.	No eukaryotic cell lines were used.
c. Report whether the cell lines were tested for mycoplasma contamination.	No eukaryotic cell lines were used.
d. If any of the cell lines used are listed in the database of commonly misidentified cell lines maintained by ICLAC, provide a scientific rationale for their use.	No eukaryotic cell lines were used.

Animals and human research participants

Policy information about studies involving animals; when reporting animal research, follow the ARRIVE guidelines

11. Description of research animals

Provide all relevant details on animals and/or animal-derived materials used in the study.	Testes: mouse strain C57BL/6J

Policy information about studies involving human research participants

12. Description of human research participants

Describe the covariate-relevant population characteristics of the human research participants.	No human participants were involved.
